# EIT-guided end-expiratory pressure individualization in robotic surgery with high PEEP ventilation: a prospective observational study

**DOI:** 10.1186/s12871-026-03739-6

**Published:** 2026-03-10

**Authors:** Lorenz L. Mihatsch, Anja Gnadl, Fiona Harzer, Stefan Rederlechner, Patrick Friederich

**Affiliations:** 1 Technical University of Munich, Germany, TUM School of Medicine and Health, TUM University Hospital, Munich, Germany; 2 Department of Anaesthesiology, Intensive Care Medicine, and Pain Therapy, München Klinik Bogenhausen, Munich, Germany

**Keywords:** Electrical impedance tomography, EIT, Individualized PEEP, Lung-protective ventilation, Robotic surgery, High PEEP, Dynamic compliance, Driving pressure, Mechanical power, Recruitability

## Abstract

**Background:**

Pneumoperitoneum and extreme positioning during robotic surgery impose substantial mechanical load on the respiratory system. Whether bedside electrical impedance tomography (EIT) is beneficial for individualized positive end-expiratory pressure (PEEP) titration under routinely high-PEEP ventilation remains uncertain. We assessed the feasibility and benefits of EIT-guided PEEP titration during clinical routine and explored whether pre-EIT respiratory parameters or anthropometric variables can predict EIT-guided best PEEP and EIT endpoints: lung collapse reduction, overdistention reduction, and regional ventilation delay reduction in routine perioperative care.

**Materials and methods:**

Prospective observational study in 177 patients undergoing elective robotic procedures spanning steep Trendelenburg (e.g., prostate/rectal) and non-Trendelenburg (e.g., adrenalectomy) positions with a laparoscopic pressure of 20 cmH_2_O. After establishing pneumoperitoneum and final positioning, EIT (OD–CL crossing-point method) was used to titrate PEEP. Primary endpoints were EIT-guided best PEEP and change in PEEP. Secondary endpoints were change in dynamic compliance (Cdyn), driving pressure (DP), and mechanical power (MP) normalized to Cdyn (MP_adj_), as well as EIT-derived endpoints: lung collapse reduction, overdistention reduction, and regional ventilation delay reduction. Associations between pre-EIT variables and outcomes were tested by correlations and ROC analysis.

**Results:**

For the primary endpoints, EIT-guided PEEP increased pre-EIT PEEP from 12.0 ± 2.0 to 14.8 ± 2.1 cmH_2_O and, hence, by 2.8 ± 2.3 cmH_2_O. Of the secondary endpoints, Cdyn improved in 82% of the patients after EIT-guided PEEP titration. DP lowered in 31% of the patients, with post-EIT DP of  ≤ 15 cmH_2_O. Despite mechanical gains, MP_adj_ increased on average from 0.42 to 0.43 J·cmH_2_​O·min^−1^ml^−1^. Concerning the EIT-derived endpoints, lung collapse reduction was 88%, overdistention reduction was 12%, and regional ventilation delay reduction was 30%. Proportions in secondary endpoints did not differ across surgical subgroups. Baseline anthropometrics showed poor predictive value for EIT-guided best PEEP (strongest correlation with MP_adj_: r = 0.33, R^2^ = 0.109) and change in PEEP (strongest correlation with the difference between laparoscopic pressure and PEEP (lap. pressure-PEEP), r = 0.38; R^2^ = 0.144). Pre-EIT Cdyn best signalled lung collapse reduction with an ROC-derived cutoff  < 45.75 ml/H_2_O (accuracy 0.82, sensitivity 0.84, specificity 0.68).

**Discussion:**

EIT-guided PEEP titration improved ventilation parameters in a heterogeneous robotic cohort with high abdominal pressure subject to high-PEEP, maintaining lung-protective ventilation. Pre-EIT respiratory data and anthropometric variables were insufficient for the reliable prediction of EIT-guided best PEEP. Our study demonstrates that EIT can be implemented in clinical routine and allows for individualization of respiratory treatment during robotic surgery, subject to high-PEEP ventilation.

**Clinical trial number:**

Not applicable.

**Supplementary Information:**

The online version contains supplementary material available at 10.1186/s12871-026-03739-6.

## Introduction

Postoperative pulmonary complications (PPC), including atelectasis, pneumonia, and prolonged hypoxemia, are strongly associated with increased hospital length of stay, mortality, and healthcare costs [1]. These complications arise from multifactorial changes to the respiratory system, including anesthesia-induced atelectasis [2], which may be reversed by the application of positive end-expiratory pressure (PEEP) [3]. PPCs have stimulated several large multicenter studies to identify preventive measures such as high PEEP combined with low tidal volumes [4–6]. So far, the study results are ambiguous regarding interventional success [4, 5, 7], possibly resulting from a lack of individualization of PEEP settings [8–11]. It has therefore been hypothesized that PEEP needs to be individualized [8–11].

Impaired lung function by atelectasis formation may also result from pneumoperitoneum and steep Trendelenburg position [2, 12–15]. This is frequently the case in laparoscopic and robotic surgery [16]. Anaesthetic agents and muscle relaxation [13] synergistically contribute to impair lung function and atelectasis by facilitating a cranial shift of the diaphragm [12].

Accumulating evidence demonstrates that electrical impedance tomography (EIT) enables individualized PEEP titration, primarily via the overdistension-collapse (OD-CL) crossing-point method. This technique optimizes PEEP by identifying the equilibrium point that minimizes both alveolar collapse and overdistension. The OD-CL crossing-point approach [17, 18] has been validated in diverse surgical contexts [19, 20]. EIT-guided PEEP improves lung mechanics in abdominal/robotic surgeries [21, 22]. This is attributed to EIT’s ability to detect and reverse dorsal lung collapse [23]. Additionally, during robotic-assisted laparoscopy in steep Trendelenburg’s position and beyond, EIT-guided PEEP individualization improves ventilation distribution and reduces driving pressure [23] and enables real-time assessment of regional ventilation [24, 25]. Given the compounded respiratory challenges from pneumoperitoneum and extreme positioning, low-PEEP approaches seem physiologically untenable in high-abdominal-pressure settings [12, 26]. Some published comparisons use low-PEEP control arms in high-abdominal-pressure settings that warrant higher PEEP, thereby potentially disadvantaging controls and possibly overestimating EIT effects [26]. The additional benefit of EIT on top of routine high-PEEP strategies adjusted to anthropometric variables such as body mass index (BMI) is currently unanswered, with unknown consequences to concepts of lung protective ventilation.

Routine robotic surgery involves high fixed laparoscopic insufflation and thus intra-abdominal pressures of 20 cmH_2_O. It is therefore conceivable to counteract this laparoscopic pressure (lap. pressure) by high PEEP levels, as routine practice in our institution. Whether additional bedside EIT-guided PEEP individualization produces consistent, clinically relevant improvement in ventilator settings and respiratory mechanics remains uncertain. It is also unclear if readily available pre-EIT ventilation parameters already anticipate the required PEEP adjustment and whether simple patient characteristics meaningfully influence EIT-derived decisions. However, establishing EIT in clinical routine is challenging since it is technically demanding and due to a lack of standardization in data processing and interpretation of the analysis [27, 28]. These results would therefore be a prerequisite for establishing EIT in perioperative routine, in addition to protective ventilation parameters readily available from common ventilators, dynamic compliance (Cdyn) [29, 30], and mechanical power (MP) [31, 32].

The objectives of our study, therefore, were:

1. To quantify the additional effect of EIT-guided PEEP titration on routine ventilator settings and lung mechanics in patients undergoing a broad range of robotic procedures with routinely high PEEP and lung protective ventilation, characterized by a diverse range of body positionings.
2. To examine (a) associations between anthropometric and ventilation parameters before EIT-guided PEEP optimization with the EIT-guided best PEEP and change in PEEP, as well as (b) their influence on the change in standard EIT parameters, i.e., lung collapse reduction, overdistention reduction, and regional ventilation delay reduction.

## Material methods

### Study design and study population

This prospective observational study involved seven different types of transperitoneal robotic surgeries, each characterized by a distinct patient positioning with routinely applied high-PEEP ventilation. The study period was from 10/2022 to 03/2025.

### Ethics

Patients gave written and informed consent to participate in this study prior to inclusion. Ethical approval was waived by the ethical committee of the Bayerische Landesärztekammer (Ref. 2020–1255) since all data collected were routine and observational in nature and, thus, fall under the provisions of the Bavarian Hospital Act (BayKrG, Art. 27). The study adheres to the Declaration of Helsinki and its later amendments. Clinical trial number was not applicable.

### Detectable effect size

No a priori sample size calculation was performed. Given the achieved sample (*N* = 177), we estimated the minimum detectable effect sizes at 80% power (two-sided α = 0.05) for key analyses: (i) Pearson correlation, minimal detectable r ≥ 0.29, and (ii) paired mean comparisons, minimal detectable Cohen’s d ≥ 0.21. Subgroup analyses are exploratory, and the corresponding *P*-values are descriptive.

### Anaesthesia

Balanced anaesthesia consisted of fentanyl or sufentanil, the induction agent propofol, and the muscle relaxant atracurium or rocuronium. Anaesthesia was maintained with sevoflurane, isoflurane, or desflurane, unless the patient had a history of postoperative nausea and vomiting, in which case total intravenous anaesthesia with propofol via target-controlled infusion was used. The choice of anaesthetic induction, maintenance, and intraoperative muscle relaxation was performed as per the department’s standard procedures and at the discretion of the anaesthesiologist. Neuromuscular blockade was monitored using train-of-four stimulation. Lung-protective ventilation was delivered, targeting a tidal volume (TV) of 6 ml/kg per predicted body weight (PBW), a driving pressure (DP, see below) < 15 cmH_2_O, and a high PEEP set at the anaesthesiologist’s discretion (mean 11.98 ± 1.99 cmH_2_O).

The driving pressure (DP) was calculated as DP = P_insp_—PEEP, where the inspiratory pressure (P_insp_) and PEEP were both set at the Dräger Perseus® A500 (Dräger Medical, Lübeck, Germany) ventilator in pressure-controlled mode. Static compliance (Cstat) was calculated by Cstat = TV/DP. The dynamic compliance (Cdyn), which accounts for continuously changing signals of pressure, flow, and tidal volume [30], was automatically calculated by the ventilator using a manufacturer-implemented algorithm. While the exact computational details are proprietary, an extensive description can be found elsewhere [29, 30].

### Electrical impedance tomography measurement

EIT was performed using the PulmoVista® 500 system (Dräger Medical, Lübeck, Germany) after induction of anaesthesia and establishment of the pneumoperitoneum. Patients were positioned either supine with a 30° Trendelenburg or anti-Trendelenburg tilt, or in lateral decubitus with 45° flexion. The EIT belt was placed at the 4th–5th(6th) intercostal space according to manufacturer recommendations. Ventilation was delivered in pressure-controlled mode, followed by a decremental PEEP trial starting at 20–22 cmH_2_O and reduced in 2 cmH₂O steps with at least 10–15 respiratory cycles to a minimum of 6–8 cmH_2_O. Optimal PEEP was identified using the OD-CL crossing-point method [28, 33], referring to relative changes in overdistention and relative lung collapse [34]. Signal quality was confirmed before each manoeuvre by verifying electrode contact impedance and signal-to-noise ratio. The EIT device was synchronised with the ventilator, enabling parallel acquisition of ventilation parameters. Final interpretation of the OD–CL curves and the clinically applied PEEP was performed by the attending anaesthesiologist trained in EIT interpretation. The EIT-derived target PEEP was rounded to the nearest integer value.

### Statistical analysis

All analyses were performed in R version 4.3.3 (The R Foundation for Statistical Computing, Vienna, Austria). Categorical variables are summarized as counts and percentages. Continuous variables are presented as mean ± standard deviation (SD), as well as median and interquartile range (IQR). There is no missing data contained in this analysis.

Between-group comparisons across procedure types used Kruskal–Wallis tests with Holm-adjusted pairwise Wilcoxon rank-sum tests when appropriate. Categorical variables were compared using Pearson’s χ^2^ test or Fisher’s exact test with simulated P-values based on 10,000 replications, as appropriate. MP was calculated by$$MP\left[{min}^{-1}\right]=0.098\;\times$$$$Respiratory Rate \left[{min}^{-1}\right]\times\;Tidal\;Volume\;[L]\times (PEEP\left[cmH2O\right]+\;$$$$\Delta {P}_{insp}[cmH2O])$$ [31, 32] and was normalized to Cdyn as a correlate of lung size [35, 36], i.e., $${MP}_{adj}[J\times cmH2O\times {min}^{-1}\times {ml}^{-1}]=MP/Cdyn$$.          

Associations between pre-EIT ventilation parameters with post-EIT best PEEP and with change in PEEP, as well as with EIT-derived endpoints: lung collapse reduction, overdistention reduction, and regional ventilation delay reduction, were quantified using Pearson’s or biserial correlation coefficients, as appropriate, and linear regression models.

Receiver-operating-characteristic (ROC) analysis with Youden’s J statistic was used to determine an optimal cutoff of pre-EIT Cdyn [29, 30] and the pre-EIT Cstat for classifying lung collapse reduction; performance is reported as accuracy, sensitivity, and specificity with 95% confidence intervals. All reported P-values are two-sided, and a threshold of *p* < 0.05 was considered statistically significant.

## Results

A total of 215 patients were screened for eligibility. Of these, 17 patients had no informed consent, 11 had technical difficulties with EIT, and 10 had incomplete data recordings. Consequently, 177/215 (82.3%) patients were enrolled and included in the final analysis. Patient characteristics of the 177 patients included are shown in Table 1.

**Table 1:** Patient characteristics

Anthropometric Characteristic	All *N* = 177^*1*^
Sex	
Male	106/177(60%)
Female	71/177(40%)
Height [cm]	174 ± 9 (174; 168–180)
Weight [kg]	80 ± 18 (78; 67–92)
Age [y]	63 ± 14 (64; 56–73)
BMI [kg/m^2^]	26.5 ± 5.2 (25.7; 22.9–28.7)
PBW [kg_PBW_]	72 ± 8 (73; 66–78)
**Surgical Procedures Performed**	
Adrenalectomy	26/177 (14.7%)
Fundoplication or Hiatoplasty	16/177 (9.0%)
Hemicolectomy	15/177 (8.5%)
Kidney Surgery	31/177 (17.5%)
Sigmoid or Rectal Resection	31/177 (17.5%)
Prostate Surgery	38/177 (21.5)
Other Robotic Surgery Procedures^2^	20/177 (11.3)
**Baseline Parameters**	
SpO_2_ [%]	98.21 ± 1.59 (99.00; 98.00–99.00)
FiO_2_ [%]	0.49 ± 0.11 (0.46; 0.40–0.54)
etCO_2_ [mmHg]	37.5 ± 5.4 (37.0; 34.0–41.0)
TV [ml]	419 ± 73 (419; 367–470)
TV_PBW_ [ml/kg_PBW_]	5.83 ± 0.93 (5.84; 5.28–6.42)
Resp. Rate. [1/min]	15.63 ± 1.63 (15.00; 15.00–17.00)
PEEP [cmH_2_O]	11.98 ± 1.99 (12.00; 10.00–14.00)
DP [cmH_2_O]	12.33 ± 1.67 (12.00; 11.00–14.00)
Lap. Pressure [cmH_2_O]	19.45 ± 1.90 (19.95; 19.95–19.95)
Lap. Pressure—PEEP [cmH_2_O]	7.47 ± 2.72 (7.95; 5.95–9.95)
Cdyn [ml/H_2_O]	40 ± 12 (39; 32–45)
Cstat [ml/H_2_O]	35 ± 9 (33; 29–39)
MP [J/min]	15.6 ± 3.6 (15.0; 13.0–18.0)
MP_adj_ [J H2O min^−1^ml^−1^]	0.42 ± 0.14 (0.40; 0.33–0.51)

*Abbreviations*: *PBW* predicted body weight, *BMI* body mass index, *TV* tidal volume, *DP* driving pressure, *PEEP* positive end-expiratory pressure, *Cdyn* dynamic compliance, *Cstat* static compliance, *MP* mechanical power, *MP*_*adj*_ mechanical power normalized to Cdyn

^1^n/N(%); Mean ± SD (Median; 25%−75%)

^2^The group comprised patients who received anastomosis, transverse colectomy, submucosal tumour resection, or TAPP hernia repair, with fewer than 10 patients per group

**Fig. 1:**
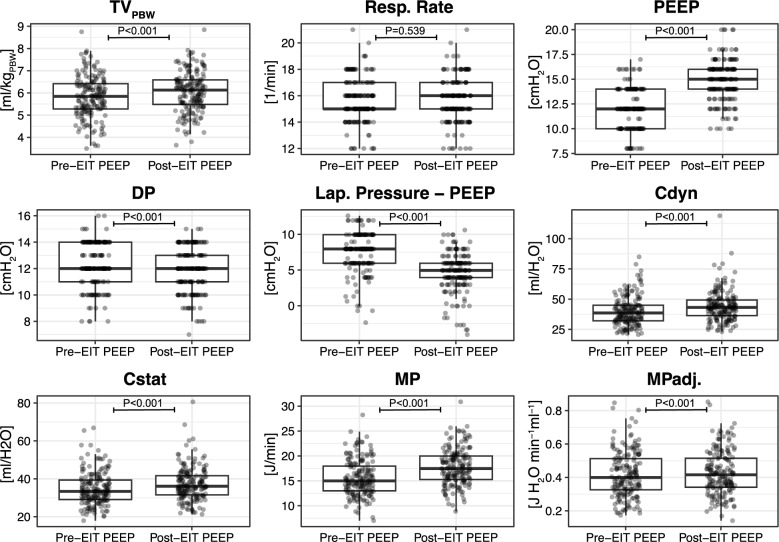
EIT-guided change in respiratory mechanics before and after PEEP titration. P-values indicate the paired Wilcoxon signed rank test with continuity correction. Points are jittered horizontally for better readability. Abbreviations: TV_PBW_ – tidal volume per predicted body weight, PEEP – positive end-expiratory pressure, DP – driving pressure, Cdyn – dynamic compliance, Cstat – static compliance, MP – mechanical power, MP_adj_ – mechanical power normalized to Cdyn

Pre- and post-EIT ventilation settings of all patients are presented and compared in Fig. 1 and Suppl. Table S1. All parameters changed significantly, except for the respiratory rate (15.63 ± 1.63/min pre-EIT and 15.66 ± 1.61/min post-EIT) and lap. pressure (19.45 ± 1.90 cmH_2_O pre-EIT and 19.48 ± 1.88 cmH_2_O post-EIT) (Fig. 1, Suppl. Table S1). Detailed analyses of pre- and post-EIT ventilation settings compared between the surgical groups are provided in Suppl. Tables S2 and S3.

### EIT-guided best PEEP and EIT-guided change in PEEP

**Fig. 2:**
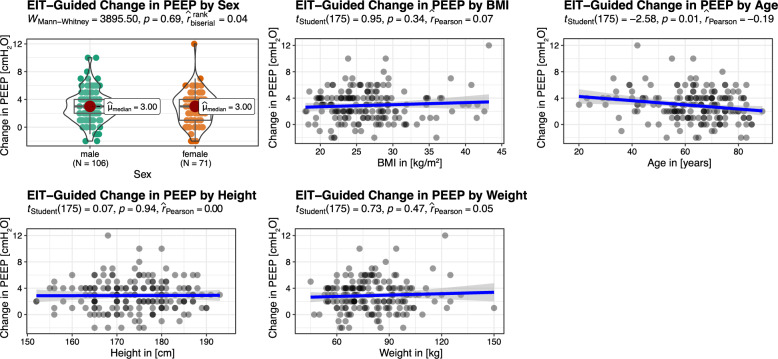
EIT-guided change in PEEP by anthropometric variables: sex, body mass index (BMI), age, and body height

Guided by EIT and the OD-CL crossing-point method, the overall PEEP was adjusted from a pre-EIT PEEP of 11.98 ± 1.99 (12.00; 10.00–14.00) cmH_2_O to post-EIT best PEEP of 14.82 ± 2.05 (15.00; 14.00–16.00) cmH_2_O. Thus, the change in PEEP was 2.89 ± 2.32 (3.00; 1.00–4.00) cmH_2_O. In 17/177 (10%) patients, the PEEP remained unchanged, whereas in 151/177 (85%) patients the PEEP was increased, and in 9/177 (5%) patients it was decreased (Table 2). There was no significant difference between the change in PEEP and the type of surgery (Suppl. Figure S1), nor between the change in PEEP and anthropometric variables, except for age. Here, a weak correlation was found (*r* = −0.19, *P* = 0.010) (Fig. 2).

**Table 2:** Percentage of patients with EIT-induced optimization of lung-protective ventilation

Characteristic	All *N* = 177^*1*^
Change in PEEP	
Decreased	9/177(5.1%)
Unchanged	17/177(9.6%)
Increased	151/177(85%)
Lung Collapse reduction	155/177(88%)
Overdistention reduction	21/177(12%)
Regional ventilation delay reduction	53/177(30%)
Cdyn improved	146/177(82%)
Cstat improved	143/177 (80%)
DP lowered	54/177(31%)
MP lowered	32/177(18%)
MP_adj_ lowered	62/177(35%)

*Abbreviations*: *PEEP* positive end-expiratory pressure, *Cdyn* dynamic compliance, *Cstat* static compliance, *DP* driving pressure, *MP* mechanical power, *MP*_*adj*_ mechanical power normalized to Cdyn

^1^ n/N(%)

**Fig. 3:**
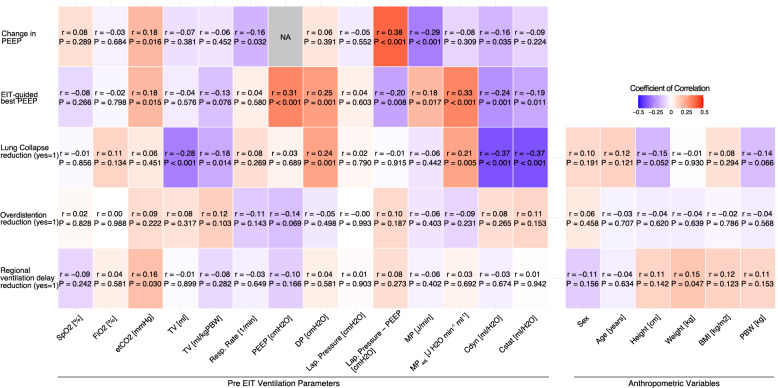
Associations between pre-EIT ventilation parameters and anthropometric variables, and primary and EIT-derived endpoints. Pearson correlations (r) are shown for continuous outcomes: Change in PEEP and EIT-guided best PEEP, and point-biserial correlations (r) for binary EIT-derived endpoints: lung collapse, overdistention, and regional ventilation delay reduction

Correlation analysis revealed weak-to-moderate, statistically significant associations between etCO_2_, PEEP, DP, lap. pressure-PEEP, MP, MP_adj_, and Cdyn pre EIT and the EIT-guided best PEEP. For the change in PEEP, correlation analysis identified weak-to-moderate, statistically significant associations with etCO_2_, respiratory rate, lap. pressure-PEEP, MP, and Cdyn (Fig. 3), with lap. pressure-PEEP showing the strongest coefficient of correlation (*r* = 0.38, *P* < 0.001). Thus, a linear regression model using the difference between lap. pressure and PEEP before EIT PEEP-optimization as a predictor explained 14.4% of the variance in the change in PEEP (R^2^ = 0.144). For the EIT-guided best PEEP, the strongest association involved MP_adj_ prior to the EIT-guided PEEP optimization (*r* = 0.33, *P* < 0.001), and the corresponding linear model explained 10.9% of the variance (R^2^ = 0.109).

### Effect of EIT-guided change in PEEP on dynamic compliance, driving pressure, and mechanical power

The magnitude of change in Cdyn, induced by the EIT-guided PEEP optimization, showed no significant associations with either of the anthropometric variables. Overall, the Cdyn after EIT-guided PEEP optimization has improved in 146/177 (82%) patients (Table 2).

After EIT-guided PEEP optimization, the change in DP was not associated with either of the anthropometric variables. Overall, DP decreased in 54/177 (31%) (Table 2), regardless of the surgical group (Suppl. Table S4). The DP after EIT PEEP optimization was ≤ 15 cmH_2_O for all patients.

Prior to EIT best PEEP, MP_adj_ did significantly differ by surgery type (*P* < 0.001), with patients receiving prostate surgery exposed to the highest MP_adj_. After EIT-guided PEEP adjustment, MP_adj_ decreased in 62/177 (35%) patients (Table 2), with no differences observed between surgery types (Suppl. Table S4). However, the overall mean of MP_adj_ increased from 0.42 ± 1.4 J·cmH_2_​O·min^−1^ml^−1^ to 0.43 ± 1.3 J·cmH_2_​O·min^−1^ml^−1^ after EIT-guided PEEP optimization. The MP_adj_ after change in PEEP did not correlate with either of the anthropometric variables.

### Prediction of EIT parameters: lung collapse, overdistention, and regional ventilation delay reduction

**Fig. 4:**
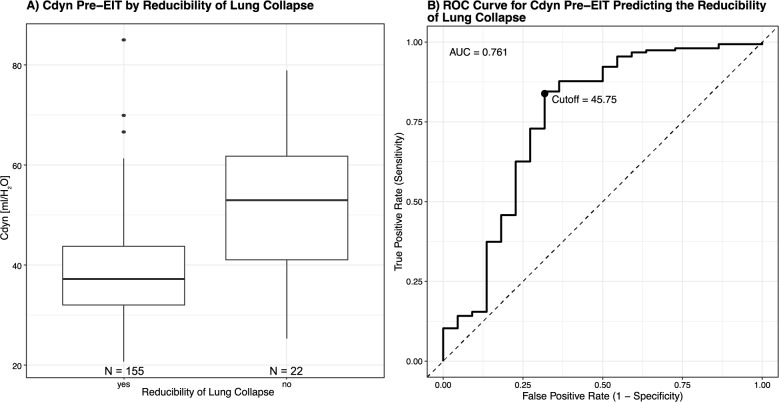
**A** Boxplot of the pre-EIT dynamic compliance (Cdyn) in ml/cmH_2_O depending on the lung collapse reducibility. **B** Receiver operating curve (ROC) for predicting lung collapse reducibility using the pre-EIT Cdyn in ml/cmH2O. The Youden index was used to find the optimal cutoff value. In comparison, an ROC-derived cutoff of the static compliance (Cstat) to classify lung collapse reduction yielded an accuracy of 0.85 (95% CI 0.780–0.892), a sensitivity of 0.871 (135/155), a specificity of 0.636 (14/22), and an optimal cutoff value of < 41.77 ml/H_2_O as well as an AUC of 0.755

Overall, lung collapse reduction was high (155/177, 88%) (Table 2). Overdistention was reduced in 12% (21/177), and regional ventilation delay in 30% (53/177). Across surgical groups, none of these proportions differed significantly between the surgical groups (lung collapse reduction: *P* = 0.556; overdistention reduction: *P* = 0.069; regional ventilation delay reduction: *P* = 0.473) (Suppl. Table S4). Neither of the three EIT parameters significantly correlated with and was, thus, not predictable by any of the anthropometric variables. Similarly, Overdistention reduction was not predictable by any of the ventilation parameters prior to EIT-guided PEEP optimization. Lung collapse reduction, however, significantly correlated with TV (r = −0.28, *P* < 0.001), TV per kg_PBW_ (*r* = −0.18, *P* = 0.014), and DP (*r* = 0.24, *P* = 0.001) as well as Cdyn (*r* = −0.37, *P* < 0.001) (Fig. 3). This correlation was best between Cdyn and lung collapse reduction and was, thus, further analysed. The difference in Cdyn before and after EIT-guided best PEEP is visualized in Fig. 4A. Using an ROC-derived (Youden) cutoff of Cdyn to classify lung collapse reduction yielded an accuracy of 0.819 (95% CI 0.755–0.873). Sensitivity was 0.839 (130/155), and specificity was 0.682 (15/22). The best cutoff value to predict lung collapse reduction was < 45.75 ml/H_2_O (Fig. 4B).

## Discussion

This study demonstrates that individualized PEEP titration guided by EIT can be effectively integrated into routine clinical practice with additional benefit in high-PEEP conditions, including those with and without steep Trendelenburg positioning [22, 37]. Secondly, we show that neither overdistention nor regional ventilation delay reduction can reliably be predicted by baseline ventilation settings or anthropometric characteristics, whereas lower Cdyn before EIT-guided PEEP optimization identified lung collapse reducibility and yielded a pragmatic cutoff of < 45.75 ml/cmH_2_O that may help prioritize EIT use in high-PEEP conditions during robotic surgery.

Our results demonstrate that EIT-guided PEEP individualization consistently increased PEEP levels while maintaining lung-protective ventilation across all robotic surgery subgroups. These effects were observed regardless of whether patients were in a steep Trendelenburg position (e.g., during prostatectomy or rectal resections) or not (e.g., during adrenalectomy), suggesting that the ventilatory consequences of EIT-guided PEEP optimization are robust across different intraoperative conditions.

### EIT-guided best PEEP and EIT-guided change in PEEP

Baseline characteristics, including sex, BMI, height, and weight, did not significantly influence the magnitude of EIT-guided optimal PEEP adjustment, underscoring the generalizability of EIT-guided PEEP titration across a heterogeneous patient population. Though age correlated significantly with the change in PEEP, the coefficient of correlation was only 0.19. Thus, age as a predictor would only account for 3.6% (R^2^) of the variation of the change in PEEP, which seems of limited clinical relevance. Slight differences in age between different surgical procedures are most likely due to different ages of disease onset of the underlying conditions warranting surgery. Importantly, despite common clinical practice, these findings also highlight that anthropometric variables are insufficient to reliably individualize PEEP.

Further, our data also show that ventilatory baseline variables are insufficient to predict EIT individualized PEEP settings with clinically relevant accuracy. Although the difference between laparoscopic pressure and PEEP before EIT-guided optimization demonstrated the strongest correlation with the change in PEEP (*r* = 0.38), the corresponding regression model explained only 14.4% of the variance, which is too low for accurate and clinically useful predictions. Similarly, the MP_adj_ before EIT best PEEP correlated weakly with the final EIT-guided best PEEP (*r* = 0.33), and its predictive model showed even lower explanatory power, i.e., 10.9%, and large prediction errors. These findings confirm that neither the magnitude nor the direction of change in PEEP can be reliably anticipated using conventional anthropometric or ventilation parameters before EIT-guided PEEP optimization. This highlights the existing limitations of conventional baseline ventilatory monitoring and reinforces the possible additional value of tools such as EIT for individualized PEEP optimization. In summary, neither simple models nor anthropometric variables can substitute EIT for individualized PEEP titration.

### Effect of EIT-guided change in PEEP on dynamic compliance, driving pressure, and mechanical power

EIT-guided PEEP-titration improved Cdyn in most patients (82%) and decreased DP in a relevant subset (31%). This is consistent with lung collapse reduction and more favourable pressure–volume behaviour. MP normalized to Cdyn (MP_adj_​) increased only slightly, from 0.42 to 0.43 J·cmH_2​_O·min^−1^ml^−1^ after EIT-guided PEEP optimization. Prior work reported an odds ratio of 1.34 per 1 SD increase in MP_adj_​ [35]. In that dataset, 1 SD ≈ 0.215 J·cmH_2_O·min^−1^ml^−1^. Thus, a 0.01-unit change equals 0.01/0.215 = 0.046 SD, implying 1.4% higher odds of PPC. This effect size is small and likely clinically negligible. In summary, we hypothesize that EIT-guided best PEEP titration yields a small increase in MP_adj_, with a PPC risk that is likely clinically negligible, in favour of improved ventilation conditions with increased Cdyn (82%), reduced DP (31%), and reduced lung collapse (88%) in a clinically non-negligible number of patients. The net risk of PPC remains to be investigated. Changes in Cdyn, DP, and MP_adj_ were largely independent of anthropometrics. Since anthropometric variables do not change intraoperatively, this supports the view that EIT-guided PEEP, rather than fixed patient factors or the type of surgery, drives the observed improvements [38]. The relative value and hierarchy of PEEP, MP_adj._, Cdyn, and DP in optimizing lung-protective ventilation should be investigated prospectively, especially in conditions with lung pathology.

### Prediction of EIT-derived endpoints: lung collapse, overdistention, and regional ventilation delay reduction

Variables before EIT-guided PEEP optimization provided only limited predictive value for the EIT endpoints. Overdistention reduction and regional ventilation delay reduction were not predictable from anthropometrics or baseline ventilator settings. It may not be surprising that dynamic changes of ventilation conditions, as measured by EIT in real time, are not predictable by static quantities, such as anthropometric variables [38].

Reduction of lung collapse was the best signalled EIT-derived endpoint, with lower Cdyn before EIT-guided PEEP optimization associated with a higher reduction in lung collapse. As this parameter was binary, we assessed discrimination using ROC analysis. A simple Cdyn threshold (< 45.75 ml/H_2_O) yielded good performance (sensitivity 0.84; specificity 0.68; balanced accuracy 0.76). Thus, low Cdyn can flag likely lung collapse reduction, whereas a negative test cannot exclude it. In other words, Cdyn was the best single cue before EIT-guided PEEP optimization we identified, but it is not sufficient as a stand-alone decision rule. The analysis based on static compliance (Cstat) yielded the same conclusions.

A pragmatic pathway could be to use Cdyn to prioritize EIT when resources are constrained. When the baseline Cdyn is high, the likelihood of reducible lung collapse is low, and further PEEP increase is more likely to drive overdistention and mechanical power without meaningful benefit. In resource-limited settings, such cases can be deprioritized for EIT-guided ventilatory adjustment. Further prospective, procedure-stratified studies should verify this “differential indication” approach and quantify its impact on workflow and outcomes. Still, Cdyn is a global measure. Thus, it cannot localize collapse or overdistension nor detect regional ventilation delay. Hence, selective EIT remains valuable to confirm minimal effective PEEP, avoid overdistension, and reduce regional ventilation delay.

Approximately 12% of the screened patients were excluded due to technical problems or incomplete EIT recordings, posing a non-negligible barrier to the routine clinical implementation of EIT. Common contributors include competing workflow demands, limited availability of devices, materials, and expertise, as well as inconsistent data interpretability [27]. This introduces potential selection bias and underscores the need for pragmatic countermeasures, including standardized analysis and parameter reference values, backup devices and materials, and a user-friendly standard operating procedure [27]. It may also include a fallback to standard-of-care ventilation or other concepts of ventilation optimization [39], such as the recruitment-to-inflation ratio [40], when EIT is unavailable.

Taken together, these findings reinforce that regional EIT imaging is needed to determine the reducibility of lung collapse, mitigate overdistention, and reduce regional ventilation delay intraoperatively. Future work should evaluate multivariable models that incorporate dynamic/mechanical signals (e.g., stepwise PEEP responses, plateau pressures, EIT-derived global inhomogeneity/regional compliance indices) and should undergo external validation and decision-analytic assessment to clarify clinical utility beyond simple thresholds, particularly in robotic surgery [16].

### Strengths and limitations

This study has several notable strengths. It is one of the largest real-world investigations to date, examining the application of EIT-guided PEEP individualization in a heterogeneous cohort undergoing robotic surgery subject to high-PEEP ventilation. The prospective collection of high-resolution ventilation and hemodynamic data allowed for a detailed physiological assessment before and after EIT-guided PEEP titration. Importantly, we included a broad range of surgical procedures, including both steep Trendelenburg and non-Trendelenburg positions, thereby enhancing the generalizability of our findings across surgical contexts. Furthermore, subgroup analyses by procedure type were adequately powered to detect medium to large physiological effects, enabling clinically meaningful conclusions for specific patient groups.

However, some limitations must be acknowledged. Firstly, the study was conducted at a single academic centre, potentially limiting generalizability to other perioperative settings with different ventilatory strategies or institutional protocols, such as those employing lower baseline PEEP strategies [12]. In lower-PEEP settings, EIT is expected to increase PEEP to reduce lung collapse [12], which is in line with our results. This effect may even be higher if baseline PEEP is low. Consequently, regardless of the baseline PEEP, EIT serves to individualize PEEP, not to uniformly escalate it: Either to reduce lung collapse, or to reduce overdistention, or to optimize the tradeoff between lung collapse and overdistention. Secondly, EIT-guided PEEP optimization was performed at a single standardized intraoperative time point after pneumoperitoneum and positioning. Therefore, potential time-dependent changes in respiratory mechanics during prolonged procedures were not captured, and the optimal PEEP may evolve over the course of surgery [38]. Thirdly, EIT availability and occasional electrode malfunctions are well-known problems for the implementation of EIT in routine clinical care [27] and restricted enrolment in this study, potentially biasing our results. Fourthly, although we included a wide variety of robotic procedures, the subgroups still represent a limited sample size for multivariable modelling, particularly regarding the prediction of EIT-individualized PEEP settings and reducibility of lung collapse. Fifthly, despite robust statistical methodology, the correlational nature of the study design limits causal inference. The absence of long-term clinical outcomes, such as PPC, precludes conclusions regarding the prognostic impact of EIT-guided PEEP optimization. Consequently, any inference about reductions in PPC risk remains speculative, and the findings should be considered hypothesis-generating. Future studies should include standardized PPC endpoints and longitudinal follow-up to test whether physiological benefits translate into improved clinical outcomes. Nevertheless, this large real-world study demonstrates that EIT can be implemented in routine clinical practice.

## Conclusion

Across a broad spectrum of robotic procedures and patient positionings with application of high PEEP, EIT allowed for individualized PEEP optimization and reduced lap. pressure–PEEP, with overall improvements in DP and Cdyn. Regarding prediction, anthropometric variables and baseline ventilator settings showed only weak associations with the EIT-guided best PEEP and the change in PEEP, indicating the limited utility of model-based targeting of individualized best PEEP. For EIT endpoints, overdistention reduction and regional ventilation delay reduction were not predictable from baseline parameters, whereas lower Cdyn before EIT-guided PEEP optimization identified higher reducibility of lung collapse.

Collectively, these findings support EIT as a practical tool for individualized intraoperative ventilator management and argue against one-size-fits-all settings, while highlighting that ventilatory setting parameters prior to EIT-guided PEEP optimization alone are insufficient to replace individualized EIT-guided ventilation.

## Supplementary Information

Supplementary Material 1: Supplementary Figure S1. EIT-guided change in PEEP by type of surgery. Supplementary Table S1. Ventilation Settings Pre- and Post-EIT PEEP Optimization. Supplementary Table S2. Patient Characteristics and Pre-EIT Setting Changes by Type of Surgery. Supplementary Table S3. Post-EIT Ventilation Setting Changes by Type of Surgery. Supplementary Table S4. Percentage of Patients with EIT-Induced Optimization of Lung-Protective Ventilation Across Surgical Groups.

## Data Availability

All data generated or analysed during this study are included in this published article and its supplementary information files.
