# Supplementary material for: EIT-guided end-expiratory pressure individualization in robotic surgery with high PEEP ventilation: a prospective observational study
Source: BMC Anesthesiol. 2026 Mar 10;26:219. doi: 10.1186/s12871-026-03739-6 (PMC13064359; doi:10.1186/s12871-026-03739-6)
Supplement: Supplementary file 1 — Supplementary Material 1: Supplementary Figure S1. EIT-guided change in PEEP by type of surgery. Supplementary Table S1. Ventilation Settings Pre- and Post-EIT PEEP Optimization. Supplementary Table S2. Patient Characteristics and Pre-EIT Setting Changes by Type of Surgery. Supplementary Table S3. Post-EIT Ventilation Setting Changes by Type of Surgery. Supplementary Table S4. Percentage of Patients with EIT-Induced Optimization of Lung-Protective Ventilation Across Surgical Groups. [file 12871_2026_3739_MOESM1_ESM.docx]

**Supplementary Figure S1.** *EIT-guided change in PEEP by type of surgery*

**Supplementary Table S1.** *Ventilation Settings Pre- and Post-EIT PEEP Optimization*

| **Characteristic** | **Pre-EIT PEEP Optimization** | **Post-EIT PEEP Optimization** | **P-Val.**^2^ |
| --- | --- | --- | --- |
| TV_PBW_ [ml/kg_PBW_] | 5.83±0.93 (5.84; 5.28-6.42) | 6.00±1.00 (6.13; 5.45-6.57) | <0.001 |
| Resp. Rate [1/min] | 15.63±1.63 (15.0; 15.0-17.0) | 15.66±1.61 (16.00 15.0-17.0) | 0.539 |
| PEEP [cmH_2_O] | 11.98±1.99 (12.0; 10.0-14.0) | 14.82±2.05 (15.0; 14.0-16.0) | <0.001 |
| DP [cmH_2_O] | 12.33±1.67 (12.0; 11.0-14.0) | 11.91±1.69 (12.0; 11.0-13.0) | <0.001 |
| Lap. Pressure - PEEP [cmH_2_O] | 7.47±2.72 (7.95; 5.95-9.95) | 4.65±2.72 (4.95; 3.95-5.95) | <0.001 |
| Cdyn [ml/H_2_O] | 40±12 (39; 32-45) | 44±13 (43; 36-49) | <0.001 |
| Cstat [ml/H_2_O] | 35±9 (33; 29-39) | 37±9 (36; 32-42) | <0.001 |
| MP [J/min] | 15.6±3.6 (15.0; 13.0-18.0) | 17.7±3.6 (17.5; 15.3-20.0) | <0.001 |
| MP_adj_ [J H2O min^-1^ml^-1^] | 0.42±0.14 (0.40; 0.33-0.51) | 0.43±0.13 (0.42; 0.34-0.51) | <0.001 |
| ^1^ n/N(%); Mean±SD (Median; 25%-75%).  *^2^* Paired Wilcoxon signed rank test with continuity correction.  Abbreviations: **PBW** – predicted body weight, **TV** – tidal volume, **DP** – driving pressure, **PEEP** – positive end-expiratory pressure, **Cdyn** – dynamic compliance, **Cstat** – static compliance, **MP** – mechanical power, **MP_adj_** – mechanical power normalized to Cdyn. | | | |

**Supplementary Table S2.** *Patient Characteristics and Pre-EIT Setting Changes by Type of Surgery. All surgical subgroups were balanced in terms of sex, except for the group of patients receiving prostate surgery. Patients of the latter group were significantly taller (P = 0.011) and had significantly higher PBW (P = 0.001), reflecting male exclusiveness. Patients receiving adrenalectomy or fundoplication were significantly younger (P = 0.001), reflecting the early onset of underlying disease. Compared to the other patient groups, patients undergoing prostate surgery in a steep Trendelenburg position were ventilated with a significantly lower TV per kgPBW (P = 0.010), higher respiratory rate (P = 0.046), higher PEEP (P = 0.031), and higher DP (P = 0.003) prior to EIT measurement. With the laparoscopic pressure not significantly different between groups (P = 0.366), the difference between lap. pressure and PEEP (Lap. Pressure-PEEP) was significantly smaller in the prostate surgery group (P = 0.010), and dynamic compliance was significantly worse (P = 0.003).*

| **Characteristic** | **All** N = 177*^1^* | **Adrenalectomy** N = 26*^1^* | **Fundoplicatio/ Hiatoplasty** N = 16*^1^* | **Hemicolectomy right** N = 15*^1^* | **Kidney Surgery** N = 31*^1^* | **Sigma- or Rectumresection** N = 31*^1^* | **Prostate** N = 38*^1^* | **Other Robotic Surgery** N = 20*^1^* | **P-Val.***^2^* |
| --- | --- | --- | --- | --- | --- | --- | --- | --- | --- |
| **Patient Characteristics** | | | | | | | | | |
| Sex |  |  |  |  |  |  |  |  | 0.697*^3^* |
| male | 106/177(60%) | 12/26(46%) | 7/16(44%) | 8/15(53%) | 18/31(58%) | 16/31(52%) | 38/38(100%) | 7/20(35%) |  |
| female | 71/177(40%) | 14/26(54%) | 9/16(56%) | 7/15(47%) | 13/31(42%) | 15/31(48%) | - | 13/20(65%) |  |
| Height [cm] | 174±9  (174; 168-180) | 173±9  (172; 166-181) | 171±10  (169; 165-179) | 173±9  (170; 165-180) | 174±10  (175; 168-181) | 173±9  (175; 167-180) | 178±6  (178; 174-183) | 170±8  (170; 164-175) | 0.017 |
| Weight [kg] | 80±18  (78; 67-92) | 82±21  (76; 66-93) | 82±18  (88; 67-96) | 78±17  (80; 65-87) | 81±18  (80; 66-90) | 77±18  (76; 64-92) | 86±18  (84; 74-96) | 71±14  (70; 63-74) | 0.068 |
| Age [y] | 63±14  (64; 56-73) | 56±15  (58; 44-63) | 58±14  (59; 49-68) | 68±9  (67; 62-77) | 63±15  (66; 55-75) | 65±13  (66; 59-71) | 69±8  (69; 63-75) | 55±17  (57; 43-71) | 0.001 |
| BMI [kg/m^2^] | 26.5±5.2  (25.7; 22.9-28.7) | 27.2±6.6  (25.7; 22.5-29.8) | 27.8±5.5  (28.2; 24.6-31.7) | 26.1±5.7  (24.1; 22.5-29.3) | 26.5±4.4  (25.6; 23.4-29.2) | 25.7±5.2  (25.9; 20.6-28.7) | 27.0±4.8  (26.4; 24.1-28.6) | 24.8±5.1  (23.4; 21.8-25.3) | 0.344 |
| PBW [kg] | 72±8  (73; 66-78) | 71±8  (71; 65-78) | 69±8  (68; 64-75) | 72±7  (69; 66-78) | 72±8  (72; 68-78) | 72±8  (73; 66-77) | 76±6  (76; 73-80) | 68±6  (67; 63-73) | 0.001 |
| **Pre-EIT Measurement** | | | | | | | | | |
| SpO_2_ [%] | 98.21±1.59  (99.00; 98.00-99.00) | 98.62±1.36  (99.00; 98.00-100.00) | 98.25±1.61  (99.00; 98.00-99.00) | 98.13±2.26  (98.00; 97.00-100.00) | 97.94±1.36  (98.00; 97.00-99.00) | 98.85±1.39  (99.00; 98.00-100.00) | 97.89±1.64  (98.00; 97.00-99.00) | 98.16±1.61  (99.00; 97.00-99.00) | 0.122 |
| FiO_2_ [%] | 0.49±0.11  (0.46; 0.40-0.54) | 0.53±0.14  (0.49; 0.42-0.59) | 0.48±0.10  (0.46; 0.41-0.53) | 0.44±0.07  (0.44; 0.39-0.50) | 0.49±0.11  (0.45; 0.40-0.59) | 0.47±0.10  (0.45; 0.41-0.54) | 0.48±0.13  (0.45; 0.39-0.53) | 0.49±0.10  (0.50; 0.41-0.57) | 0.484 |
| etCO_2_ [mmHg] | 37.5±5.4  (37.0; 34.0-41.0) | 35.7±3.1  (35.0; 34.0-38.0) | 35.3±10.7  (37.5; 32.5-40.0) | 38.1±5.0  (38.0; 34.0-41.0) | 36.9±5.1  (36.0; 33.0-40.0) | 37.4±4.6  (37.0; 34.5-40.5) | 39.2±4.5  (39.0; 37.0-42.0) | 38.4±4.5  (38.0; 34.0-42.0) | 0.054 |
| TV [ml] | 419±73  (419; 367-470) | 433±71  (428; 374-489) | 426±76  (426; 377-469) | 412±75  (400; 360-466) | 437±72  (450; 367-486) | 408±85  (404; 350-473) | 413±81  (413; 370-452) | 404±52  (403; 355-450) | 0.576 |
| TV_PBW_ [ml/kg_PBW_] | 5.83±0.93  (5.84; 5.28-6.42) | 6.11±0.79  (6.27; 5.34-6.75) | 6.17±1.08  (6.37; 5.62-6.60) | 5.72±0.89  (5.98; 5.10-6.16) | 6.07±0.86  (6.00; 5.45-6.62) | 5.96±1.04  (6.06; 5.30-6.60) | 5.40±1.00  (5.40; 4.56-5.98) | 5.66±0.70  (5.63; 5.21-6.04) | 0.010 |
| Resp. Rate. [1/min] | 15.63±1.63  (15.00; 15.00-17.00) | 15.62±1.36  (15.00; 15.00-16.00) | 15.44±1.21  (15.00; 14.50-16.00) | 15.80±1.82  (15.00; 15.00-18.00) | 15.13±1.52  (15.00; 14.00-16.00) | 15.10±1.62  (15.00; 14.00-16.00) | 16.32±1.85  (16.00; 15.00-18.00) | 15.68±1.58  (16.00; 15.00-16.00) | 0.046 |
| PEEP [cmH_2_O] | 11.98±1.99  (12.00; 10.00-14.00) | 11.54±2.30  (10.50; 10.00-14.00) | 11.75±1.65  (12.00; 10.50-12.50) | 11.87±2.36  (12.00; 10.00-14.00) | 11.61±1.96  (12.00; 10.00-14.00) | 11.55±1.47  (12.00; 10.00-12.00) | 13.05±1.94  (12.00; 12.00-14.00) | 11.84±1.71  (12.00; 10.00-14.00) | 0.031 |
| DP [cmH_2_O] | 12.33±1.67  (12.00; 11.00-14.00) | 12.35±1.62  (12.00; 11.00-14.00) | 11.63±1.54  (12.00; 10.50-12.50) | 12.47±1.60  (12.00; 12.00-14.00) | 11.71±1.72  (12.00; 10.00-13.00) | 11.70±2.11  (12.00; 10.00-14.00) | 13.13±1.44  (14.00; 12.00-14.00) | 12.65±1.28  (12.00; 12.00-14.00) | 0.003 |
| Lap. Pressure [cmH_2_O] | 19.45±1.90  (19.95; 19.95-19.95) | 19.80±0.78  (19.95; 19.95-19.95) | 18.45±2.91  (19.95; 17.96-19.95) | 19.68±1.03  (19.95; 19.95-19.95) | 19.39±2.68  (19.95; 19.95-19.95) | 19.95±1.29  (19.95; 19.95-19.95) | 19.22±1.95  (19.95; 19.95-19.95) | 19.56±1.42  (19.95; 19.95-19.95) | 0.366 |
| Lap. Pressure - PEEP [cmH_2_O] | 7.47±2.72  (7.95; 5.95-9.95) | 8.26±2.19  (8.46; 5.95-9.95) | 6.70±3.22  (7.95; 5.95-7.95) | 7.82±2.59  (7.95; 5.95-9.95) | 7.78±3.00  (7.95; 5.95-9.95) | 8.40±2.16  (7.95; 7.95-9.95) | 6.16±2.93  (6.29; 3.96-7.95) | 7.73±2.18  (7.95; 5.95-9.95) | 0.010 |
| Cdyn [ml/H_2_O] | 40±12  (39; 32-45) | 42±11  (42; 34-45) | 47±17  (43; 36-58) | 38±9  (40; 29-46) | 44±10  (45; 34-53) | 41±14  (39; 34-45) | 35±11  (34; 29-41) | 37±8  (36; 32-40) | 0.003 |
| Cstat [ml/H_2_O] | 35±9  (33; 29-39) | 36±8  (36; 30-41) | 37±8  (36; 31-44) | 34±8  (33; 26-43) | 38±9  (38; 30-46) | 36±12  (33; 30-39) | 32±9  (31; 26-36) | 32±5  (32; 29-35) | 0.033 |
| MP [J/min] | 15.6±3.6  (15.0; 13.0-18.0) | 15.9±4.0  (14.6; 12.9-18.9) | 15.0±2.7  (14.5; 13.8-16.7) | 15.4±3.3  (15.5; 12.0-19.1) | 15.1±3.3  (15.0; 12.3-18.2) | 14.0±3.6  (14.5; 11.1-16.0) | 17.1±3.8  (16.3; 14.8-19.2) | 15.3±3.5  (14.2; 12.6-17.1) | 0.081 |
| MP_adj_ [J H2O min^-1^ml^-1^] | 0.42±0.14 (0.40; 0.33-0.51) | 0.40±0.12 (0.39; 0.33-0.51) | 0.35±0.12 (0.36; 0.23-0.44) | 0.42±0.12 (0.39; 0.33-0.46) | 0.36±0.10 (0.36; 0.28-0.43) | 0.37±0.15 (0.34; 0.28-0.42) | 0.51±0.14 (0.53; 0.41-0.59) | 0.43±0.11 (0.42; 0.36-0.55) | <0.001 |
| ^1^ n/N(%); Mean±SD (Median; 25%-75%); ^2^ Pearson’s Chi-squared test; Kruskal-Wallis rank sum test; ^3^ P-Value refers to all groups excluding prostate surgery.  Abbreviations: **PBW** – predicted body weight, **BMI** – body mass index, **TV** – tidal volume, **DP** – driving pressure, **PEEP** – positive end-expiratory pressure, **LP** – laparoscopic intraabdominal pressure, **Cdyn** – dynamic compliance, **Cstat** – static compliance, **MP** – mechanical power, **MP_adj_** – mechanical power normalized to Cdyn. | | | | | | | | | |

**Supplementary Table S3.** *Post-EIT Ventilation Setting Changes by Type of Surgery.*

| **Characteristic** | **All** N = 177*^1^* | **Adrenalectomy** N = 26*^1^* | **Fundoplicatio/ Hiatoplasty** N = 16*^1^* | **Hemicolectomy right** N = 15*^1^* | **Kidney Surgery** N = 31*^1^* | **Sigma- or Rectumresection** N = 31*^1^* | **Prostate** N = 38*^1^* | **Other Robotic Surgery** N = 20*^1^* | **P-Val.***^2^* |
| --- | --- | --- | --- | --- | --- | --- | --- | --- | --- |
| SpO_2_ [%] | 98.15±1.48  (98.00; 98.00-99.00) | 98.50±1.50  (99.00; 98.00-100.00) | 97.81±1.56  (98.00; 98.00-99.00) | 98.47±1.60  (98.00; 98.00-100.00) | 98.06±1.06  (98.00; 97.00-99.00) | 98.60±1.19  (99.00; 98.00-99.00) | 97.87±1.58  (98.00; 97.00-99.00) | 98.03±1.72  (98.00; 97.00-99.00) | 0.243 |
| FiO_2_ [%] | 0.46±0.10  (0.43; 0.40-0.50) | 0.48±0.12  (0.43; 0.40-0.53) | 0.46±0.13  (0.42; 0.40-0.49) | 0.42±0.06  (0.41; 0.39-0.46) | 0.46±0.11  (0.43; 0.38-0.57) | 0.45±0.09  (0.41; 0.39-0.54) | 0.47±0.10  (0.46; 0.40-0.50) | 0.46±0.08  (0.47; 0.40-0.51) | 0.766 |
| etCO_2_ [mmHg] | 38.7±5.9  (38.0; 36.0-42.0) | 38.1±4.3  (38.0; 35.0-41.0) | 37.3±11.9  (39.0; 33.5-40.0) | 37.1±3.2  (37.0; 35.0-39.0) | 37.8±4.4  (37.0; 36.0-40.0) | 39.0±7.1  (37.0; 34.0-42.5) | 40.6±5.0  (40.0; 37.0-44.0) | 39.2±4.6  (39.0; 36.0-42.0) | 0.154 |
| TV [ml] | 434±66  (433; 388-480) | 442±65  (451; 382-491) | 435±87  (437; 357-506) | 436±60  (431; 413-480) | 449±62  (440; 390-505) | 437±77  (447; 418-480) | 423±70  (426; 392-462) | 420±50  (412; 388-458) | 0.505 |
| TV_PBW_ [ml/kg_PBW_] | 6.00±1.00  (6.13; 5.45-6.57) | 6.25±0.68  (6.23; 5.80-6.67) | 6.27±1.05  (6.49; 5.61-6.91) | 6.09±0.86  (6.23; 5.37-6.77) | 6.25±0.78  (6.31; 5.65-6.63) | 6.09±1.81  (6.38; 5.59-7.03) | 5.54±0.87  (5.56; 4.83-6.13) | 5.89±0.74  (5.94; 5.48-6.30) | 0.002 |
| Resp. Rate [1/min] | 15.66±1.61  (16.00; 15.00-17.00) | 15.46±1.53  (15.00; 15.00-16.00) | 15.31±1.25  (15.00; 14.00-16.00) | 15.93±1.58  (15.00; 15.00-18.00) | 15.19±1.51  (15.00; 14.00-16.00) | 15.10±1.62  (15.00; 14.00-16.00) | 16.45±1.70  (16.50; 15.00-18.00) | 15.74±1.55  (16.00; 15.00-16.00) | 0.015 |
| PEEP [cmH_2_O] | 14.82±2.05  (15.00; 14.00-16.00) | 14.58±2.35  (14.50; 14.00-16.00) | 14.06±1.29  (14.00; 13.00-15.00) | 14.40±2.03  (14.00; 12.00-16.00) | 13.81±1.85  (14.00; 12.00-15.00) | 14.40±1.90  (14.00; 13.50-16.00) | 16.13±1.70  (16.00; 15.00-17.00) | 15.32±2.01  (15.00; 14.00-16.00) | <0.001 |
| DP [cmH_2_O] | 11.91±1.69  (12.00; 11.00-13.00) | 11.96±1.51  (12.00; 11.00-13.00) | 10.88±1.89  (11.00; 9.50-12.00) | 11.80±1.47  (11.00; 11.00-13.00) | 11.39±1.78  (12.00; 10.00-13.00) | 11.25±1.83  (12.00; 10.00-12.00) | 12.74±1.57  (13.00; 12.00-14.00) | 12.39±1.17  (12.00; 12.00-14.00) | <0.001 |
| Lap. Pressure [cmH_2_O] | 19.48±1.88  (19.95; 19.95-19.95) | 19.80±0.78  (19.95; 19.95-19.95) | 18.45±2.91  (19.95; 17.96-19.95) | 19.68±1.03  (19.95; 19.95-19.95) | 19.39±2.68  (19.95; 19.95-19.95) | 19.95±1.29  (19.95; 19.95-19.95) | 19.36±1.90  (19.95; 19.95-19.95) | 19.56±1.42  (19.95; 19.95-19.95) | 0.528 |
| Lap. Pressure - PEEP [cmH_2_O] | 4.65±2.72  (4.95; 3.95-5.95) | 5.22±2.16  (5.45; 3.95-5.95) | 4.39±3.29  (5.45; 2.96-6.95) | 5.28±2.35  (5.95; 3.95-7.95) | 5.59±2.66  (4.95; 3.95-7.95) | 5.55±2.11  (5.95; 4.45-6.45) | 3.22±2.95  (3.95; 1.95-4.95) | 4.24±2.46  (4.95; 2.95-5.95) | 0.003 |
| Cdyn [ml/H_2_O] | 44±13  (43; 36-49) | 46±11  (44; 40-51) | 52±17  (45; 41-63) | 43±10  (41; 34-50) | 47±9  (45; 43-55) | 50±19  (47; 42-54) | 39±11  (38; 32-45) | 40±7  (40; 36-44) | <0.001 |
| Cstat [ml/H_2_O] | 37±9(36; 32-42) | 38±8(38; 34-42) | 41±12(36; 33-48) | 38±7(38; 31-41) | 40±7(39; 33-44) | 40±12(37; 31-46) | 34±8(35; 28-39) | 34±4(34; 32-36) | 0.009 |
| MP [J/min] | 17.7±3.6  (17.5; 15.3-20.0) | 17.9±4.0  (16.3; 14.7-21.7) | 16.2±3.2  (16.1; 14.4-19.2) | 17.8±3.1  (17.1; 15.1-20.6) | 16.9±3.6  (17.0; 13.6-19.8) | 16.4±3.1  (16.6; 14.2-18.1) | 19.6±3.5  (19.5; 17.3-21.2) | 17.9±3.2  (18.1; 15.7-19.3) | 0.017 |
| MP_adj_ [J H_2_O min^-1^ml^-1^] | 0.43±0.13 (0.42; 0.34-0.51) | 0.41±0.09 (0.40; 0.35-0.46) | 0.34±0.12 (0.33; 0.25-0.44) | 0.44±0.13 (0.42; 0.37-0.53) | 0.37±0.10 (0.35; 0.28-0.45) | 0.36±0.11 (0.34; 0.30-0.39) | 0.53±0.14 (0.55; 0.38-0.64) | 0.46±0.09 (0.45; 0.39-0.55) | <0.001 |
| ^1^ Mean±SD (Median; 25%-75%); ^2^ Pearson’s Chi-squared test; Kruskal-Wallis rank sum test.  Abbreviations: **PBW** – predicted body weight, **TV** – tidal volume, **PEEP** – positive end-expiratory pressure, **DP** – driving pressure, **Cdyn** – dynamic compliance, **Cstat** – static compliance, **MP** – mechanical power, **MP_adj_** – mechanical power normalized to Cdyn. | | | | | | | | | |

**Supplementary Table S4.** *Percentage of Patients with EIT-Induced Optimization of Lung-Protective Ventilation Across Surgical Groups.*

| **Characteristic** | **All** N = 177*^1^* | **Adrenalectomy or eq.** N = 26*^1^* | **Fundoplicatio/ Hiatoplastik** N = 16*^1^* | **Hemicolectomy right** N = 15*^1^* | **Kidney Surgery** N = 31*^1^* | **Sigma- or Rectumresection** N = 31*^1^* | **Prostata** N = 38*^1^* | **Other Robotic Surgery** N = 20*^1^* | **P-Val.***^2^* |
| --- | --- | --- | --- | --- | --- | --- | --- | --- | --- |
| Change in PEEP |  |  |  |  |  |  |  |  | 0.256 |
| decreased | 9/177(5.1%) | 0/26(0%) | 1/16(6.3%) | 1/15(6.7%) | 3/31(9.7%) | 1/20(5.0%) | 2/38(5.3%) | 1/31(3.2%) |  |
| unchanged | 17/177(9.6%) | 4/26(15%) | 3/16(19%) | 0/15(0%) | 6/31(19%) | 1/20(5.0%) | 2/38(5.3%) | 1/31(3.2%) |  |
| increased | 151/177(85%) | 22/26(85%) | 12/16(75%) | 14/15(93%) | 22/31(71%) | 18/20(90%) | 34/38(89%) | 29/31(94%) |  |
| Lung Collapse reduction | 155/177(88%) | 21/26(81%) | 15/16(94%) | 13/15(87%) | 26/31(84%) | 16/20(80%) | 35/38(92%) | 29/31(94%) | 0.556 |
| Overdistention reduction | 21/177(12%) | 1/26(3.8%) | 4/16(25%) | 4/15(27%) | 5/31(16%) | 3/20(15%) | 3/38(7.9%) | 1/31(3.2%) | 0.069 |
| Regional Ventilation Delay reduction | 53/177(30%) | 5/26(19%) | 6/16(38%) | 4/15(27%) | 10/31(32%) | 5/20(25%) | 16/38(42%) | 7/31(23%) | 0.473 |
| Cdyn improved | 146/177(82%) | 20/26(77%) | 15/16(94%) | 12/15(80%) | 25/31(81%) | 16/20(80%) | 32/38(84%) | 26/31(84%) | 0.892 |
| Cstat improved | 143/177(81%) | 21/26(81%) | 14/16(88%) | 11/15(73%) | 24/31(77%) | 14/20(70%) | 31/38(82%) | 28/31(90%) | 0.577 |
| DP lowered | 54/177(31%) | 10/26(38%) | 7/16(44%) | 5/15(33%) | 9/31(29%) | 5/20(25%) | 11/38(29%) | 7/31(23%) | 0.753 |
| MP lowered | 32/177(18%) | 5/26(19%) | 4/16(25%) | 3/15(20%) | 8/31(26%) | 4/20(20%) | 7/38(18%) | 1/31(3.2%) | 0.231 |
| MP_adj_ loweres | 62/177(35%) | 9/26(35%) | 7/16(44%) | 6/15(40%) | 12/31(39%) | 9/20(45%) | 9/38(24%) | 10/31(32%) | 0.650 |
| ^1^ n/N(%); ^2^ Fisher’s Exact Test for Count Data with simulated P-value (based on 10 000 replicates).  Abbreviations: **PEEP** – positive end-expiratory pressure, **Cdyn** – dynamic compliance, **Cstat** – static compliance, **DP** – driving pressure, **MP** – mechanical power, **MP_adj_** – mechanical power normalized to Cdyn. | | | | | | | | | |
